# Dietary habits and vaginal environment: can a beneficial impact be expected?

**DOI:** 10.3389/fcimb.2025.1582283

**Published:** 2025-06-18

**Authors:** Marielle Ezekielle Djusse, Federica Prinelli, Tania Camboni, Camilla Ceccarani, Clarissa Consolandi, Silvia Conti, Margherita Dall’Asta, Francesca Danesi, Luca Laghi, Francesco Matteo Curatolo, Sara Morselli, Claudio Foschi, Paola Castellano, Antonella Marangoni, Marco Severgnini

**Affiliations:** ^1^ Section of Microbiology, Department of Medical and Surgical Sciences (DIMEC), Alma Mater Studiorum - University of Bologna, Bologna, Italy; ^2^ International PhD College, Collegio Superiore of Alma Mater Studiorum, University of Bologna, Bologna, Italy; ^3^ Institute of Biomedical Technologies, National Research Council, Segrate, Italy; ^4^ National Biodiversity Future Center S.c.a.r.l., Palermo, Italy; ^5^ Department of Medical Sciences, University of Ferrara, Ferrara, Italy; ^6^ Department of Animal Science, Food and Nutrition (DIANA), Università Cattolica Del Sacro Cuore, Piacenza, Italy; ^7^ Human Nutrition Unit, Department of Agricultural and Food Sciences (DISTAL), University of Bologna, Cesena, Italy; ^8^ Interdepartmental Centre for Agri-Food Industrial Research (CIRI Agrifood), University of Bologna, Cesena, Italy; ^9^ Centre of Foodomics, Department of Agricultural and Food Sciences (DISTAL), University of Bologna, Cesena, Italy; ^10^ Microbiology Unit, IRCCS Azienda Ospedaliero-Universitaria di Bologna, Bologna, Italy; ^11^ Department of Medical and Surgical Sciences (DIMEC), Alma Mater Studiorum - University of Bologna, Bologna, Italy

**Keywords:** vaginal microbiota, diet, macronutrients, metabolome, women’s health

## Abstract

**Introduction:**

In reproductive-aged women, a vaginal microbiota dominated by several Lactobacillus species is crucial for maintaining vaginal health. Among the various factors affecting the composition of the vaginal ecosystem, the impact of dietary habits has rarely been explored. Thus, in this cross-sectional study, we assessed the role of macronutrient intake on the vaginal microbiota in a cohort of 113 young women, independently from potential confounders.

**Methods:**

For each subject, we characterized (i) the vaginal bacterial community-state type (CST) by 16S rRNA gene profiling, (ii) the vagina lmetabolic profile by 1H-NMR spectroscopy, and (iii) the energy, nutrient and alcohol intake through a validated food frequency questionnaire.

**Results:**

We found that the increase in animal protein intake, mainly derived from red and processed meat, was positively associated with the dysbiotic condition of CST IV and, similarly, alcohol consumption was significantly associated with the levels of Gardnerella spp. and Ureaplasma spp. On the other hand, we noticed a beneficial effect of a-linolenic acid, with its increase inversely associated with CST III, dominated by the ‘less-protective’ species Lactobacillus iners. Moreover, linolenic acid was related to the abundance of Lactobacillus crispatus, in turn related tovarious vaginal metabolites such as 4-hydroxyphenyllactate and several amino acids. Total carbohydrates, vegetable proteins, total fiber, and starch were negatively correlated with Gardnerella spp.

**Discussion:**

We highlighted that specific dietary habits (i.e., reduced consumption of alcohol and animal proteins, higher intake of linolenic acid) can have a beneficial impact on the vaginal environment, through the maintenance of a microbiota mainly dominated by ‘protective’ Lactobacillus species.

## 1 Introduction

In reproductive-aged women, a protective vaginal microbiota is typically dominated by various species of *Lactobacillus*. These beneficial bacteria play a key role in maintaining vaginal health by preventing colonization and proliferation of harmful microorganisms through several mechanisms (Severgnini et al., 2022; Morselli et al., 2024).

The depletion of lactobacilli, in parallel with the increase in different species of anaerobic bacteria (e.g., *Gardnerella*, *Fannyhessea*, *Prevotella*, *Mobiluncus*), results in a dysbiotic condition called bacterial vaginosis (BV) (Ceccarani et al., 2019; Plummer et al., 2024). BV is characterized by marked alterations in the composition of vaginal metabolites (e.g., reduction of lactic acid, higher concentrations of biogenic amines, and short chain fatty acids), and is associated with an increased risk of sexually transmitted infections (STIs), as well as adverse pregnancy outcomes such as preterm birth and low birth weight (Srinivasan et al., 2015; Laghi et al., 2021; Occhipinti et al., 2025).

Molecular characterization of the vaginal microbiota using 16S rRNA gene amplicon sequencing has afforded the high-resolution characterization of the composition and taxonomy of the vaginal microenvironment (Tamarelle et al., 2024). Indeed, vaginal bacterial communities can be clustered into five broad community state types (CSTs) (Ravel et al., 2011; Greenbaum et al., 2019). CST I, II, III, and V are dominated by one species of *Lactobacillus*, *L. crispatus*, *L. gasseri*, *L. iners*, and *L. jensenii*, respectively (Ravel et al., 2011). While *L. crispatus*, *L. gasseri*, and *L. jensenii* are well-recognized as hallmarks of vaginal health, *L. iners* has often been considered a ‘transitional’ species, showing fewer protective capabilities (Raimondi et al., 2021; Novak et al., 2022).

Contrariwise, CST IV, which aligns well with BV, is depleted of *Lactobacillus* spp. and consists of a diverse array of anaerobes, including *Gardnerella* spp., *Fannyhessea vaginae (Atopobium vaginae)*, *Candidatus Lachnocurva vaginae* (formerly known as BVAB1) or *Prevotella* spp., further classifiable as CST IV-A, CST IV-B or CST-IVC (France et al., 2020).

Longitudinal studies have shown that the composition of the vaginal microbiota is influenced by various local and systemic factors, such as hormonal levels, psychosocial stress, smoking, sexual habits, the use of topical products or antibiotics, and the presence of urogenital infections (Govender and Ghai, 2024; Morsli et al., 2024).

In this context, it has also been shown that dietary habits can affect the bacterial composition of the vaginal environment (Tuddenham et al., 2019; Dall’Asta et al., 2021; Noormohammadi et al., 2022). Indeed, previous studies have demonstrated that increased dietary fat intake, energy intake, and glycemic load are associated with a higher risk of BV, whereas increased intake of folate, vitamin A, calcium, and more in general, diets rich in fiber, seem to be protective factors against this condition (Neggers et al., 2007; Thoma et al., 2011; Shivakoti et al., 2020).

Recently, in a cohort of pregnant Caucasian women, we observed that a lactobacilli-dominated vaginal microbiota was negatively correlated with a higher pre-pregnancy intake of animal-sourced proteins, whereas a higher pre-pregnancy consumption of total carbohydrates and sugars seemed to be a protective factor for vaginal health (Dall’Asta et al., 2021).

Nevertheless, exhaustive data on the impact of dietary macronutrient intake on the composition of the vaginal environment are still lacking, and many aspects remain to be fully elucidated.

Therefore, in this cross-sectional study, we assessed the role of energy, macronutrient, alcohol intake and related dietary sources on the vaginal ecosystem in terms of bacterial composition and vaginal metabolite concentration in a cohort of 113 non-pregnant young women, considering the effect of potential confounders that could perturb the vaginal environment.

To this goal, we characterized for each woman (i) the vaginal bacterial composition by means of 16S rRNA gene profiling, (ii) the vaginal metabolic profile (^1^H-NMR spectroscopy), and (iii) the dietary nutrient intake using a validated food frequency questionnaire.

## 2 Materials and methods

### 2.1 Study setting and population

Between November 2023 and April 2024, 119 sexually active women having sex with men (WSM) of reproductive age were included in the study. The women were volunteers attending degree courses at the University of Bologna, Italy, and were selected from those who expressed an interest in participating in the study. At the time of the enrollment, exclusion criteria were: (i) antibiotic use in the month prior to sampling; (ii) menstruation at the time of sampling; (iii) HIV infection; (iv) presence of chronic conditions (e.g., diabetes, autoimmune diseases, malignancies); (v) pregnancy at enrollment and/or previous pregnancies; (vi) age < 18 years. Moreover, women with urogenital infections due to sexually transmitted pathogens (i.e., *Chlamydia trachomatis*, *Neisseria gonorrhoeae, Trichomonas vaginalis, Mycoplasma genitalium*) were further excluded when diagnostic tests results were available.

The following data and variables were recorded for each woman: age, education (categorized as high school, university degree, and postgraduate/doctorate), work status (student vs. other), marital status (married/cohabiting vs. single/separated), contraceptive use (yes vs. no), smoking habits (never, former, or current smoker), body mass index (BMI, calculated as weight in kg divided by height in cm squared), and presence/absence of vaginal symptoms or disorders (i.e., dyspareunia, vaginal itching and/or burning, and unusual vaginal discharge) (see **Table 1**).

**Table 1:** Descriptive characteristics of the study population stratified by CST (n = 113).

		CST I – II-V (n=61, 54%)		CST III (n=33, 29.2%)		CST IV (n=19, 16.8%)		*p*	Total (n=113)	
Anthropometric, behavioral and clinical parameters										
Age (median, IQR)*		21	3	20	3	20	3	.367	21	3
Education (n, %)	High school degree	38	62.30	22	66.67	12	63.16	0.868	72	63.72
	University degree or PhD/residency	16	26.23	9	27.27	6	31.58		31	27.43
	Na	7	11.48	2	6.06	1	5.26		10	8.85
Work position (n, %)	Student	49	80.33	31	93.94	16	84.21	0.340	96	84.96
	Other	5	8.20	0	0.00	2	10.53		7	6.19
	Na	7	11.48	2	60.06	1	5.26		10	8.85
Marital status (n, %)	Married/cohabitant	7	11.48	4	12.12	2	10.53	0.880	13	11.50
	Single/separated	47	77.05	27	81.82	16	84.21		90	79.65
	Na	7	11.48	2	6.06	1	5.26		10	8.85
Hormonal contraception use (n, %)		15	24.59	8	24.24	6	31.58	.810	29	25.66
Smokers (n, %)		15	24.59	6	18.18	8	42.11	.157	29	25.66
BMI (mean, SD)		20.96	3.67	20.83	3.88	21.80	2.02	.593	21.26	3.15
Dyspareunia (n, %)		20	32.79	7	21.21	1	5.26	.045	28	24.78
Vaginal itching and/or burning (n, %)		8	13.11	7	21.21	1	5.26	.267	16	14.16
Unusual vaginal discharge (n, %)		6	9.84	6	18.18	3	15.79	.491	15	13.27
PSS scale (median, IQR)		22	8	21	6	21	8	0.884	21	7
Microbiota α-diversity (median, IQR)*										
Shannon		4.41	1.32	5.33	2.18	6.70	0.97	<.001	4.85	2.23
Chao1		422.53	229.21	417.90	271.81	628.25	331.93	.037	447.67	317.30
Observed species		218	186	278	252	449	210	<.001	257	275
PD whole tree		4.74	2.60	4.56	2.68	5.95	2.78	.364	4.97	2.59
Alcohol intake (n, %)										
Abstainers		6	9.84	2	6.06	1	5.26	.588	9	7.96
≤ 1 AU per day		50	81.97	27	81.82	14	73.68		91	80.53
> 1 AU per day		5	8.20	4	12.12	4	21.05		13	11.50
Daily macronutrient intake										
Total proteins (% mean, SD)		15.70	2.18	15.14	2.89	16.68	3.32	.126	15.70	2.64
Animal proteins (% mean, SD)		10.62	2.82	9.83	3.72	11.54	3.70	.187	10.54	3.28
Vegetal proteins (% mean, SD)		5.72	1.06	5.86	1.24	5.82	1.21	.839	5.78	1.13
Total fats (% mean, SD)		39.33	4.89	36.79	3.74	38.02	6.10	.053	38.37	4.90
Saturated fatty acids (% mean, SD)		12.41	2.12	11.65	2.39	11.76	2.20	.227	12.08	2.23
Monounsaturated fatty acids (% mean, SD)		19.00	3.32	17.89	2.39	18.42	4.14	.279	18.58	3.25
Linoleic fatty acid (% mean, SD)		4.93	1.29	4.51	0.73	4.84	1.19	.224	4.79	1.14
Linolenic fatty acid (% mean, SD)		0.45	0.17	0.37	0.08	0.44	0.14	.034	0.43	0.15
Other polyunsaturated fatty acids (% mean, SD)		0.18	0.11	0.20	0.11	0.15	0.10	.373	0.18	0.11
Carbohydrates (% mean, SD)		44.97	5.70	48.07	5.07	45.29	5.51	.032	45.93	5.62
Starch (% mean, SD)		27.34	5.59	29.52	5.71	27.60	6.21	.205	28.02	5.76
Simple sugars (% mean, SD)		19.35	5.03	20.19	4.39	19.43	5.16	.718	19.61	4.85
Fibre intake (g mean, SD)		22.65	9.55	22.52	9.15	22.77	10.53	.996	22.633	9.52
Energy intake (Kcal mean, SD)		2011.6	756.4	1942.1	445.8	1915.0	525.4	.800	1975.1	639.7
MEDI-LITE (g mean, SD)		11.39	2.25	11.94	2.18	11.68	2.64	.541	11.60	2.29

For each CST and the total column, the two numbers shown represent the values as indicated for each variable in parentheses: median and interquartile range (IQR), number and percentage (n, %), mean and standard deviation (SD), percentage (mean ± SD), grams (mean ± SD), or kilocalories (mean ± SD), depending on the variable.

AU, alcoholic unit. ANOVA test and Kruskal-Wallis (*) were used to compare continuous variables, whereas Chi-squared test was employed to compare categorical variables. Na, not available.

As previously reported, marital status was used as a proxy for sexual behavior (Exavery et al., 2015; Duberstein Lindberg and Singh, 2008; Jackson et al., 2019). The Perceived Stress Scale (PSS) was used to assess the level of stress and discomfort perceived by participants during the past month (available for 103 participants out of 113). The scale includes 10 items, and the answers are provided on a 5-point Likert scale (from 0 = Never to 4 = Very often) (Cohen et al., 1983).

Each woman completed a validated food frequency questionnaire to assess dietary habits (see specific paragraph) and underwent two self-collected vaginal samples during the late follicular phase. The first swab (E-swab, Copan, Brescia, Italy) was used for diagnostic tests to exclude the presence of sexually-transmitted infections using nucleic acid amplification tests (Alinity m STI Assay; Abbott Molecular Diagnostics, Des Plaines, IL, USA). The second was collected with a sterile cotton bud, resuspended in 1 mL of sterile saline, and stored at -80°C until use. Frozen vaginal swabs were thawed, vortexed for 1 min, and removed from the liquid. After centrifugation (10000 × *g* for 15 min), cell-free supernatants were used for metabolomic analysis, whereas bacterial pellets were employed for vaginal microbiota profiling (see paragraphs below).

Written informed consent was obtained from all participants, and the study protocol was approved by the Bioethics Committee of the University of Bologna (protocol number 0122421).

### 2.2 Vaginal microbiota molecular profiling

Nucleic acids were extracted from vaginal swabs using the DNeasy Blood & Tissue kit (QIAGEN GmbH, Hilden, Germany), following the manufacturer’s protocol.

The V3-V4 hypervariable regions of the bacterial 16S rRNA gene were amplified according to the 16S metagenomic sequencing library preparation protocol (Illumina, San Diego, CA, USA). Final indexed libraries were prepared by equimolar (4 nmol/L) pooling, denaturation, and dilution to 6 pmol/L before loading on one MiSeq flow cell (Illumina) for a 2 × 300 bp paired-end run. Raw sequence data were processed by rebuilding single amplicon fragments from the paired reads using PANDAseq (Masella et al., 2012) and quality-filtering the obtained fragments trimming from the 3’-end stretches of at least three bases with a Phred score < 3; trimmed reads shorter than 75% of the original fragment were discarded. The retained reads were clustered in zero-radius Operational Taxonomic Units (zOTUs) using usearch (version 11.0.667; Edgar, https://www.biorxiv.org/content/10.1101/081257v1) and taxonomic classification was performed using the RDP classifier against SILVA 138 release (Wang et al., 2007; Quast et al., 2013). Depth of sequencing was set to the lowest sequenced sample (n=6,627 reads), in order to compensate for the sequencing unevenness of the samples and to provide a consistent minimum amount for the downstream analysis, carried out through the QIIME pipeline (version 1.9.0; Caporaso et al., 2010). Alpha-diversity evaluation was performed according to several microbial diversity metrics (i.e., Chao1, Shannon Index, Observed Species, and the Faith’s phylogenetic tree diversity metric), Beta-diversity evaluated using unweighted and weighted UniFrac distances (Lozupone et al., 2011) and the principal coordinate analysis (PCoA) to graphically represent the data.

*Lactobacillus* species-level characterization was performed as in Severgnini et al. (2022) by BLAST-aligning all reads belonging to the *Lactobacillaceae* family to a custom reference database made up of all available reference sequences in the NIH-NCBI database (https://ftp.ncbi.nlm.nih.gov/genomes/GENOME_REPORTS/prokaryotes.txt) of 17 species commonly found in the vaginal environment and filtering the results in order to obtain an unequivocal classification. In case of multiple matches with the same confidence, the taxonomy was reset to “Unclassified *Lactobacillus*”. The custom database included a total of 3,392 genomes at different finishing grades.

Community-state types (CST) of the vaginal microbial communities were determined from the taxonomic profiles using VALENCIA, a nearest centroid-based tool that classifies samples into 5 major CST and 13 sub-CST according to the similarity to a set of about 13,000 reference microbial profiles (France et al., 2020).

### 2.3 Vaginal metabolome assessment

Metabolomic analysis was performed by ^1^H-NMR spectroscopy: 100 μL of a D_2_O solution of 3-(trimethylsilyl)-propionic-2,2,3,3-d4 acid sodium salt (TSP) 10 mM set to pH 7.0 was added to 700 µL of the cell-free supernatants of the vaginal swabs. ^1^H-NMR spectra were recorded at 298 K with an AVANCE III spectrometer (Bruker, Milan, Italy), operating at a frequency of 600.13 MHz, equipped with Topspin software (version 3.5, Bruker) (Foschi et al., 2018). The signals originating from large molecules were suppressed by a CPMG filter of 400 spin-echo periods, generated by 180° pulses of 24 μs separated by 400 μs (Ventrella et al., 2016). To each spectrum, line broadening (0.3 Hz) and phase adjustment were applied by Topspin software, while any further spectra processing, molecules quantification, and data mining steps were performed in R computational language (version 4.0.5) through in-house developed scripts. The spectra were aligned toward the TSP signal, set at −0.017 ppm in agreement with Chenomx software data bank (version 8.3, Chenomx Inc., Edmonton, Alberta, Canada), and then baseline-adjusted by means of peak detection according to the “rolling ball” principle implemented in the “baseline” R package (Liland et al., 2010). The signals were assigned by comparing their chemical shift and multiplicities with the Chenomx software data bank. Molecules were quantified in the first sample acquired by employing the added TSP as an internal standard. To compensate for differences in sample amounts any other sample was then normalized to such sample by means of probabilistic quotient normalization (Dieterle et al., 2006). Integration of the signals was performed for each molecule following rectangular integration.

### 2.4 Dietary assessment and data processing

Participants’ usual dietary habits over the past one-year period were assessed using a semi-quantitative Food Frequency Questionnaire (FFQ). This FFQ was originally developed for the European Prospective Investigation into Cancer and Nutrition (EPIC) study (Bingham et al., 2001) and later validated in the Italian population (Pala et al., 2003). FFQ represents a suitable tool for evaluating long-term dietary habits, even in the context of the vaginal environment (Noormohammadi et al., 2025). The questionnaire consisted of 248 questions covering 188 different food items. Trained researchers administered the FFQ and collected data on the frequency of consumption and on portion sizes. For each food item, participants indicated their consumption frequency (per day, week, month, or year) and selected portion sizes through pictures showing small, medium, and large portions. Additional quantifiers were available, such as “smaller than the small portion”, and predefined standard portions were used when images were unavailable. Daily intake for each food item was calculated in grams, and energy, macronutrient, and alcohol intakes were derived using the Italian Food Composition Database (Gnagnarella P, Salvini S, Parpinel M. Food Composition Database for Epidemiological Studies in Italy. Version 1.2015. Available from: http://www.bda-ieo.it/).

We screened the data for implausible energy intake (<500 or >3500 kcal/day), although no participants were excluded on this basis. Alcohol intake was converted to alcoholic units (AU), with 1 AU defined as 10 g of alcohol (World Health Organization, 2000). Participants were classified according to their consumption as abstainers, those consuming ≤1 AU/day, or those consuming >1 AU/day, following national guidelines (SINU, 2024; available at: https://sinu.it/larn/). Four participants with missing dietary data were excluded from analysis.

The 188 individual FFQ food items were aggregated into 24 food categories according to their nutrient compositions and food similarities. These categories comprised tubers, vegetables, legumes, fruits, dried fruits and seeds, milk and yogurt, cheese, cereals and derived products (pasta and cereals), bakery products (bread, bread substitutes, breakfast cereals, and biscuits), stuffed pasta, sweets and snacks, red and processed meat, white meat, fish, eggs, olive oil, other plant-derived fats, animal-derived fats, sugar, non-alcoholic beverages, alcoholic beverages, vegetable products (plant-based milk and meat substitutes), salt and spices, and other non-classified foods.

To identify the main food sources of energy, macronutrients and alcohol, we conducted Spearman’s correlations analysis between all food groups and key nutritional components. These components included total proteins (TP), animal-derived protein (AP), plant-derived protein (VP), total fats (TF), total carbohydrates (TC), total dietary fiber (TDF), starch (ST), simple sugars (SS), saturated fatty acids (SFA), monounsaturated fatty acids (MUFA), linoleic acid (LA), alpha-linolenic acid (ALA), other polyunsaturated fatty acids (PUFA), alcohol, and total energy intake.

The Mediterranean diet adherence score (MEDI-LITE; Sofi et al., 2017) was calculated to provide an overall assessment of diet quality. This score ranges from 0 (minimum adherence) to 18 (maximum adherence) and evaluates the nine dietary components. The scoring system considers the consumption of whole grains, legumes, fruits, vegetables, nuts, and olive oil as positive contributors to the score, whereas the consumption of dairy products and red and processed meat are considered negative contributors. Alcohol consumption was scored according to intake levels, with moderate consumption receiving higher scores.

### 2.5 Dietary data analysis using the CoDA approach

To take into account the compositional nature of dietary data, i.e. that they are parts of a whole and essentially convey relative information, macronutrient intakes were analyzed using the Compositional Data Analysis (CoDA) approach, based on log-ratio transformations (Pawlowsky-Glahn et al., 2015). The application of this methodology to dietary data and the discussion of the interpretative implications of using different log-ratios as explanatory variables in regression models have been extensively studied (Leite and Prinelli, 2017; Corrêa Leite, 2019; Leite, 2020; Corrêa Leite, 2021). Here, we considered a nine-part macronutrient composition: AP, VP, ST, SS, SFA, MUFA, LA, ALA, PUFA.

We were interested in assessing the effect of the proportions of each component in relation to all others (relative dominance). The *pivot balance* approach is generally used for this purpose (Hron et al., 2012). A *pivot balance* can capture all relevant information regarding a compositional part. Taking advantage of the fact that the additive log-ratio (ALR) transformation and the simplified *pivot balance* are explanatory equivalents (Coenders and Pawlowsky-Glahn, 2020), we used this simple log-ratio transformation to obtain the same measures of effects as those provided by the *pivot balances*. The ALR was calculated by dividing each macronutrient by an arbitrarily chosen component (Corrêa Leite, 2021). In the present work, eight ALRs were calculated by dividing each macronutrient by starch. Each ALR represents new variables that can be included as predictor variables in standard regression models.

Let y be the outcome (CST):


$$\begin{array}{c} f(y)=B_{0}+B_{1}\times ln\frac{AP}{ST}+B_{2}\times ln\frac{VP}{ST}+B_{3}\times ln\frac{SS}{ST}+B_{4}\times ln\frac{SFA}{ST}+B_{5}\times ln\frac{MUFA}{ST}+B_{6}\\ \times ln\frac{LA}{ST}+B_{7}\times ln\frac{ALA}{ST}+B_{8}\times ln\frac{Other\;PUFA}{ST}+\sum_{i}Bi\times z_{i} \end{array}$$

where *z_i_* are the control variables.

A *B* coefficient represents the change in response associated with a one unit change in the corresponding log-ratio, holding all other log-ratios constant. The coefficient *B_1_*, for example, represents the expected change in CST when AP increases and ST decreases, while keeping all the other terms in the model constant. This necessarily means that VP, SS, SFA, MUFA, LA, ALA, and other PUFA will decrease as well by the same factor as ST. To estimate the relative dominance of ST, another set of eight ALRs was calculated with AP as the denominator and the regression model was then run a second time (Corrêa Leite, 2021).

### 2.6 Statistical analysis

Sample characteristics were described using mean and standard deviation (SD) or median and interquartile range (IQR) for continuous variables and frequencies and percentages for categorical variables. One-way ANOVA (for normally distributed variables) and Kruskal-Wallis (for non-normally distributed variables) tests were used to compare participant characteristics in relation to the three categories of CST for continuous variables and the chi-squared test was used for categorical variables. Statistical evaluation of alpha-diversity indices was performed by non-parametric Monte Carlo-based tests through the QIIME pipeline. Beta-diversity differences were assessed by a permutation test with pseudo F-ratios using the “adonis” function from R package “vegan” (version 2.0-10, https://cran.r-project.org/package=vegan). Indicator species analysis (Dufrene and Legendre, 1997) was performed in MATLAB (version 2008b, Natick, MA, USA). Macronutrient intake and metabolite concentrations were correlated with bacterial composition by calculating the pairwise Spearman’s correlation coefficients among macronutrients, metabolites and bacterial genera present ≥1% in at least one sample. Correlations were presented using heatmaps and a correlation network created using Cytoscape (version 3.10.2; Shannon et al., 2003).

Multinomial logistic regression models were constructed to estimate the independent association between the nine-parts macronutrient composition (exposure) and the three-level CST (outcome, see below), and the β-coefficient, standard error (SE), and p-value were reported. The univariate regression model included the nine-parts macronutrient composition and total energy, fiber, and alcohol intake. The multivariate model was adjusted for confounders selected on the basis of previous literature and a number of theoretical assumptions regarding their possible influence on vaginal bacterial composition, including age, marital status, BMI, and hormonal contraceptive use (Morsli et al., 2024). In addition, as psychological distress has been reported to influence vaginal microbiota (Song et al., 2020) and dietary intake (Gonçalves et al., 2024), we performed a sensitivity analysis by including the PSS scale (Cohen et al., 1983) in the model to assess whether this factor could influence the associations between macronutrient intake and CST.

For the present analysis, as CST III (*L. iners*-dominated microbiota) has been associated with vaginal dysbiosis and less protective capabilities (Mikhanoshina and Priputnevich, 2024), we grouped the CSTs into three categories: a) *L. crispatus*/*L. jensenii*/*L. gasseri*-dominated CSTs (I, II, and V); b) *L. iners*-dominated (CST III); and c) polymicrobial microbiota (CST IV).

All analyses were performed using IBM SPSS Statistics for Windows (version 24.0, IBM Corp., Armonk, NY, USA) and STATA (version 15, StataCorp LLC, College Station, TX). All p-values ≤ 0.05 were considered statistically significant.

### 2.7 Data availability

Raw sequencing data for this project are available in NCBI Short-Read Archive (SRA) under accession number PRJNA1188525 (https://www.ncbi.nlm.nih.gov/bioproject/PRJNA1188525). Raw metabolomic and nutritional data are displayed as **Supplementary material (see Excel Files S1 and S2)**.

## 3 Results

### 3.1 Vaginal environment composition

A total of 119 participants were initially recruited; excluding those with missing data on dietary exposure (n=4) and those with unavailable vaginal microbiota data (n=2), 113 subjects (mean age ± standard deviation: 21.5 ± 2.5; min-max: 19-30) were finally included in the present analysis (**Table 1**). The sequencing process generated a total of 9,474,380 raw reads, which led to 4,019,683 (42.1%) high-quality amplicon fragments mapped in the zOTUs. Samples were categorized in Community State Types (CST) according to their microbial composition. Overall, the bacterial composition of the samples resembled the expected for the vaginal environment, as per the *Lactobacillus* genus species, with *L. crispatus* being the most abundant species (40.7% on average), followed by *L. iners* (22.6%), *L. gasseri* (4.2%), and *L. jensenii* (3.7%); among the other genera, *Gardnerella vaginalis* and *Prevotella* spp. accounted for 6.1% and 6.0% on average, respectively, whereas *Streptococcus* spp. (2.4%), *Atopobium vaginae* (1.4%), and *Ureaplasma* spp. (1.1%) were also consistently present (**Supplementary Figure S1**).

**Figure 1:**
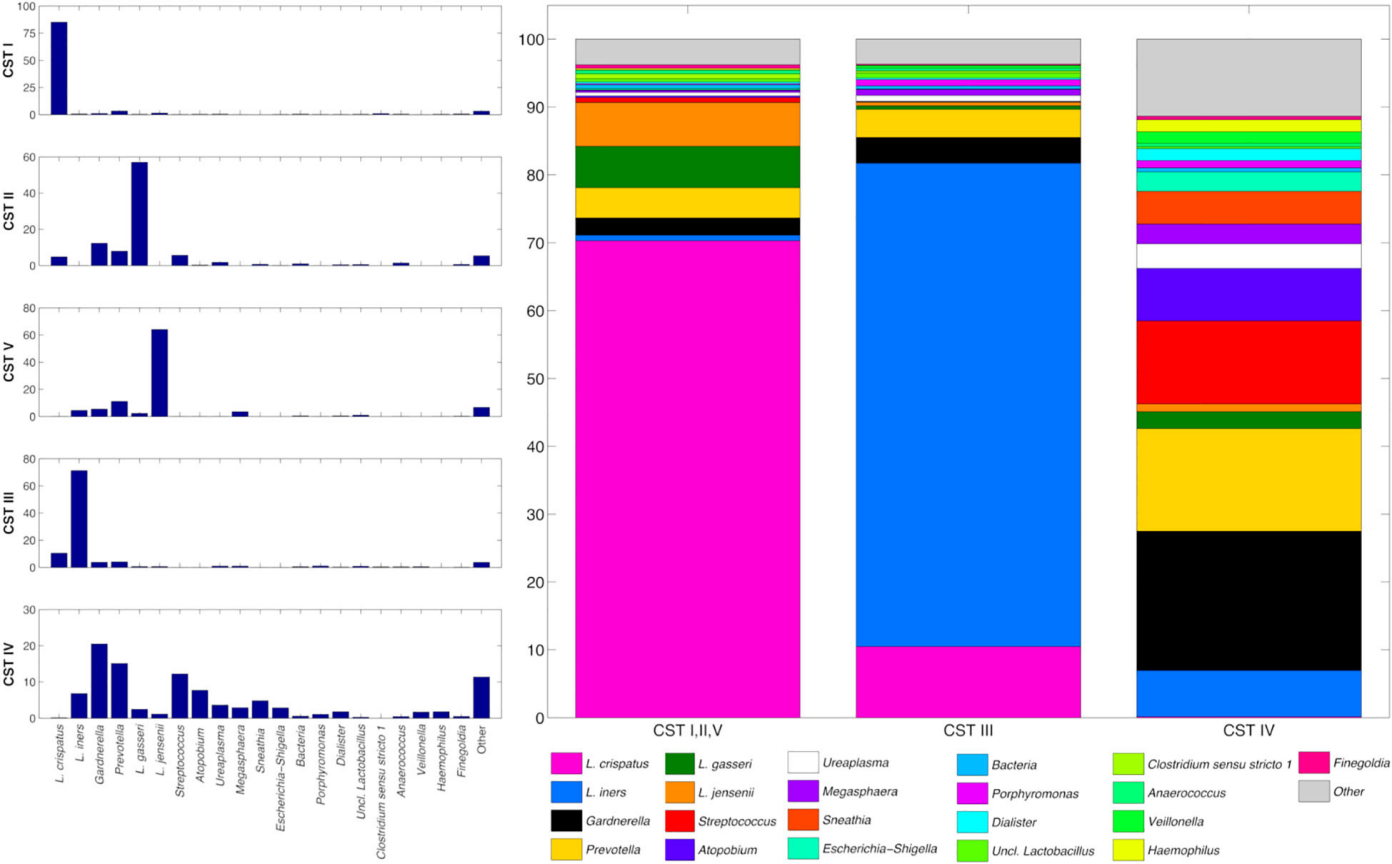
(left) Histograms of the average relative abundance (%) of bacteria over CST. (right) Stacked bars of the average relative abundance (%), grouping together samples classified in CSTs I, II and V. Only the main taxa (average abundance >0.4% over all the samples) are represented here, with *Lactobacillus* genus further subclassified to species level. Less abundant taxa are grouped in the “Other” category.

The vaginal microbiota composition was highly different among CST: CST IV had a significantly higher biodiversity compared to CST I, II, V (p=0.003) and, partially, to CST III (p=0.069), whereas no significant differences were evident between CST I, II, V and CST III. At the same time, the microbial profiles were significantly different among all the CST (p=0.001 and p=0.004 for the unweighted and the weighted UniFrac distances, respectively) (**Supplementary Figure S2**). This was reflected in the average microbial composition of the CSTs: CST I, II, V had a high prevalence of *L. crispatus*, which, nevertheless, was lower in CST III and almost absent in CST IV (70.3% vs 10.5% vs 0.2% in CST I, II, V, CST III, and CST IV, respectively). Then again, *L. iners* was a hallmark of CST III (abundance of 0.8%, 71.2% and 6.8% in CST I,II,V, CST III and CST IV, respectively), whereas the high presence of *Gardnerella vaginalis*, *Prevotella* spp., *Streptococcus* spp., *Atopobium vaginae*, and *Sneathia sanguinegens* was correlated to CST IV (accounting in total for 8.0%, 8.1%, and 60.5% in CST I,II,V, CST III and CST IV, respectively) (**Figure 1**).

Descriptive characteristics of the study population stratified by CST are displayed in **Table 1**.

### 3.2 Dietary habits

Spearman’s correlation analysis revealed distinct patterns in how different food groups contributed to macronutrient intake. For instance, for total protein consumption, significant contributors included dairy products (particularly cheese), bakery products, animal fats, and fish. Animal proteins were predominantly derived from red and processed meat, white meat, fish, and animal fats. Regarding total fat intake, the primary sources were cheese and sweets and snacks. The main sources of MUFAs were olive oils and cheese, while vegetable fats and dried fruits were the primary sources of linoleic and linolenic fatty acids, respectively. Starch intake was most closely correlated with bakery products, and total fiber intake with fruits and legumes. Total energy intake was primarily correlated with the consumption of sweets and snacks, cheese, and bakery products. Alcohol consumption showed a straightforward relationship exclusively with alcoholic beverages.

### 3.3 Association of macronutrient intake with CST

**Figure 2:**
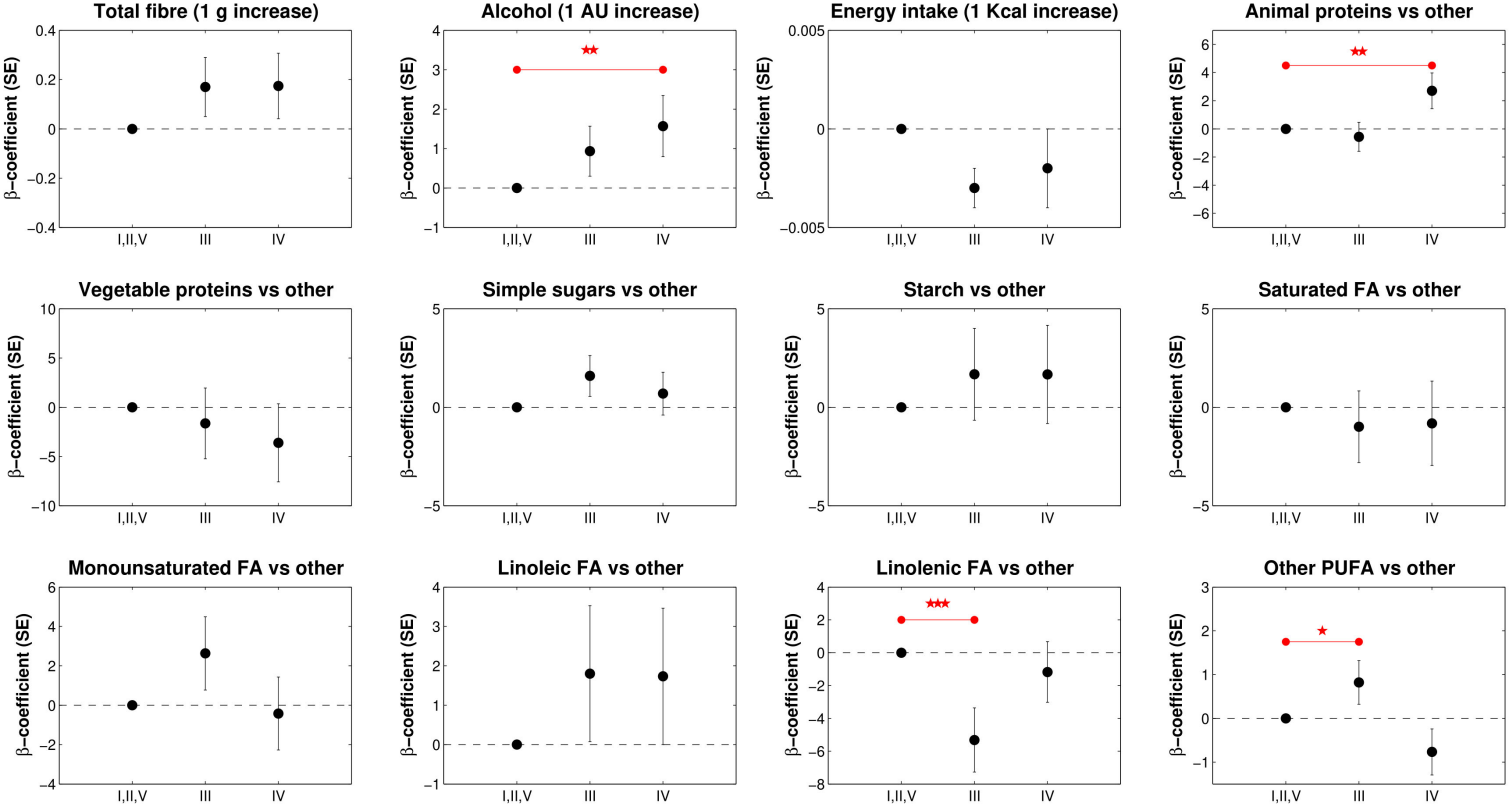
Multinomial logistic regression coefficient (b-coefficient) and standard errors (SE) of CST in relation to the macronutrient balances, total energy, fiber and alcohol intake (n=113). The model also included terms for age, BMI, marital status, and hormonal contraception use. Asterisks (*) indicate statistical significance of the model: * p<0.1; ** p<0.05, *** p<0.01.

**Figure 2** shows the results of the multivariate multinomial regression model of CST in relation to the ALR(s) transformations of macronutrients including age, marital status, BMI, and hormonal contraceptive use, total energy, fiber, and alcohol intake. We first included in the model the eight ALR(s) calculated by dividing each macronutrient by starch and then, to obtain estimates for the latter, in the second run included the ALR(s) with animal protein as the denominator. For example, the β-coefficient of “Linolenic acid vs other macronutrient” represents the negative change in CST III (β-coefficient -5.320, p-value 0.007) when linolenic FA increases and ST decreases, holding all other terms in the model constant.

In contrast, the β-coefficient of the balance “Animal proteins vs other macronutrient” expresses the positive change in CST IV (β-coefficient 2.702, *p*-value 0.033) when AP increases and ST decreases, keeping all other terms constant. We also found that an increase in alcohol units was statistically significantly positively associated with CST IV (β-coefficient 1.570, *p*-value 0.043). No other dietary components were statistically significantly associated with CSTs.

When analyzing MEDI-LITE scores across CST categories, we found no statistically significant associations (CST III β-coefficient 0.170, *p*-value 0.140; CST IV β-coefficient 0.115, *p*-value 0.395), suggesting that overall adherence to the Mediterranean diet was not associated with vaginal microbiota composition in our population (see also **Supplementary Table S1**).

With regard to psychological distress, further adjustment for this factor did not substantially alter the strength of the association between macronutrient intake and CTS (**Supplementary Table S2**).

Descriptive characteristics of the dietary macronutrients intake of the study population stratified by CST is presented in **Table 1**.

### 3.4 Correlations between macronutrients and vaginal ecosystem

**Figure 3:**
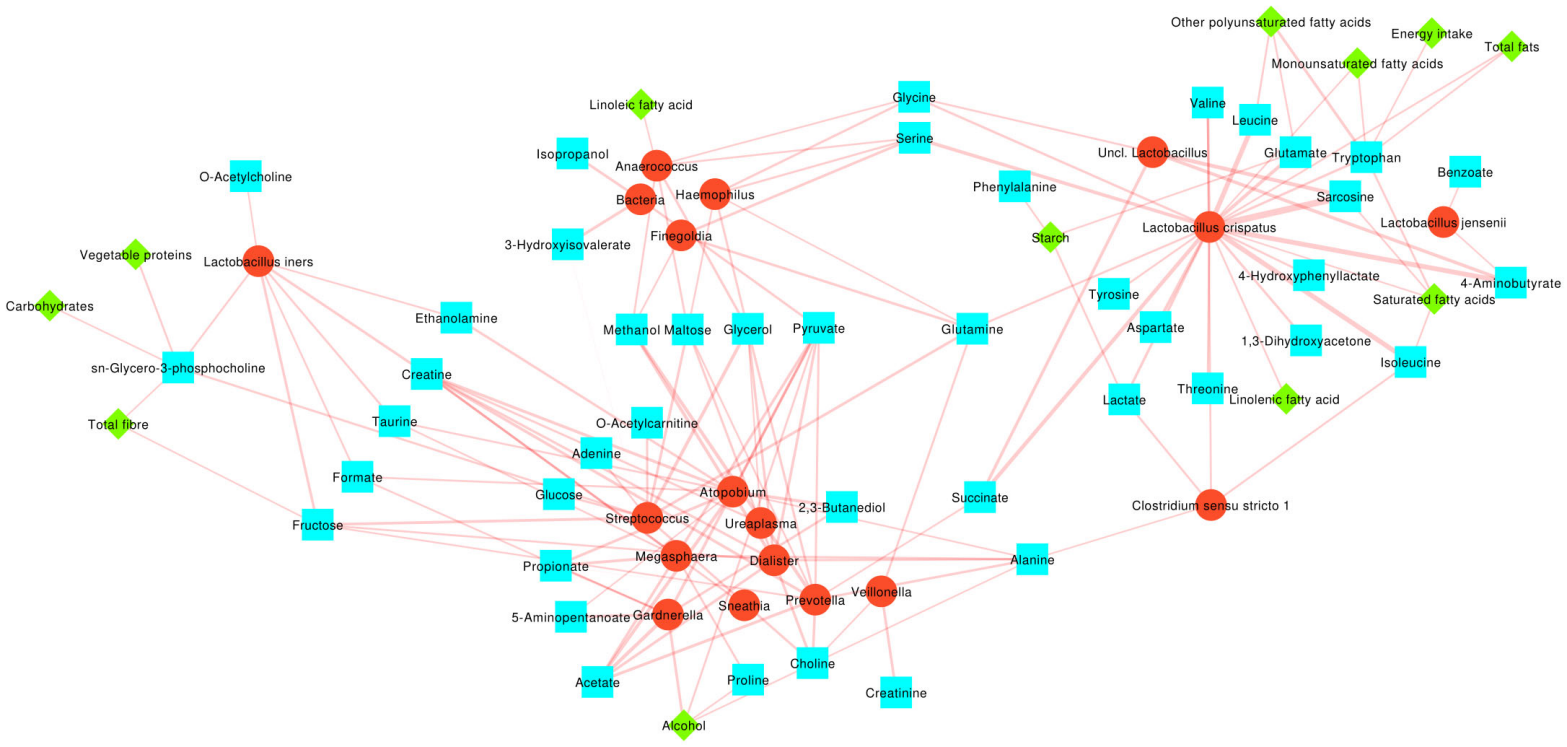
Network representing the Spearman correlation values among bacterial, metabolite and macronutrient abundances. Bacterial taxa are depicted as red circles, metabolites as cyan squares and macronutrients as green diamonds. Edges thickness is proportional to the correlation coefficient. Only significant (p<0.05) correlations are represented. Bacteria, metabolites and macronutrients are clustered according to the indicator species analysis over the three CST groups considered.

A correlation network and three pairwise heatmaps showing the Spearman correlation among bacteria, metabolites, and macronutrients were constructed in order to gain a better understanding of their mutual relationships (**Figure 3**; **Supplementary Figures S3–S5**). Bacteria were grouped according to the indicator species for the three CST groups here considered (i.e.: CST I, II, V vs. CST III vs. CST IV), with metabolites and nutrients clustered according to their significant positive correlation to the bacteria within each group.

A first group (cluster 1) was composed of *L. crispatus* (indicator species for CST I, II, V), *L. jensenii*, and other *Lactobacillus.* Several metabolites were positively associated to this group, including amino acids (i.e.: Glutamate, Leucine, Isoleucine, Valine, Tryptophan, Tyrosine, Aspartate and Threonine), their byproducts (4-Hydroxyphenyllactate, Sarcosine), 4-Aminobutyrate, Lactate, and 1,3-Dihydroxyacetone; among macronutrients, Total fats, Monounsaturated fatty acids, Other polyunsaturated fatty acids, Energy intake, Starch, and Linolenic acid all were directly or indirectly related to these group.

A second group of bacteria (cluster 2) comprised the indicator species for CST IV (e.g.: *Gardnerella, Atopobium, Megasphaera, Sneathia*, etc.). We observed several correlations to some carboxylic acids (i.e.: Creatinine, Acetate, Propionate), to amino acids (Proline, Alanine), to 2,3-Butanediol, Choline, 5-Aminopentanoate, Glucose, Adenine and O-Acetylcarnitine; interestingly, Alcohol consumption was the only macronutrient correlated to this group.

A third cluster of bacteria (cluster 3) involved those showing no differences among the CSTs (i.e.: *Finegoldia, Anaerococcus, Haemophilus, Uncl. Bacteria*) which were related to 3-hydroxyisovalerate, isopropanol, and Linoleic fatty acid.

Finally, *L. iners* formed itself a group (cluster 4), together with O-Acetylcholine and its precursor sn-Glycero-3-posphocholine, Vegetable proteins, Carbohydrates and Total fiber.

At the same time, some metabolites were common among two or more clusters: Ethanolamine, carboxylic acids such as Creatine and Formate, Taurine, and Fructose were shared between clusters 2 and 4; Methanol, carbohydrates such as Maltose and Glycerol and Pyruvate between clusters 2 and 3; amino acids such as Glycine and Serine between clusters 1 and 3; Succinate between 1 and 3; finally, Glutamine was correlated to bacteria in clusters 1, 2 and 3.

## 4 Discussion

The aim of the study was to assess in detail the relationship between dietary habits, in terms of macronutrient intake, and the taxonomic and metabolomic composition of the vaginal environment in a cohort of non-pregnant young women.

For this purpose, a total of 113 reproductive-aged women were asked to complete a validated food frequency questionnaire, while their vaginal ecosystem was analyzed both in terms of bacterial profiles (16S rRNA sequencing) and metabolic signatures (^1^H-NMR spectroscopy).

At first, we confirmed the strict association between the composition of vaginal bacterial communities (i.e., CSTs) and the levels of metabolites detected in the vaginal fluids.

Among the beneficial metabolites related to CST I (i.e.: dominated by *L. crispatus*), 4-hydroxyphenyllactate, a byproduct of amino acid degradation, has already been shown to decrease ROS (reactive oxygen species) production in both mitochondria and neutrophils, acting as a natural antioxidant (Beloborodova et al., 2012). Moreover, *Lactobacillus* species in a healthy vaginal environment are known producers of branched-chain amino acids, such as leucine, isoleucine, serine, and tryptophan (Marangoni et al., 2021). According to research, *Lactobacillus* species, including *L. crispatus*, can metabolize tryptophan into a variety of bioactive compounds, including indole derivatives. These metabolites are known to play roles in immune modulation and inflammation reduction: for instance, the metabolite norharman, produced from tryptophan by *Lactobacillus*, has been found to inhibit inflammatory responses both *in vitro* and *in vivo* (Zhou et al., 2023).

It is well known that *L. crispatus* produces high quantities of lactate, responsible for the protective acidic pH preventing pathogen proliferation in the vaginal environment, from glucose conversion through lactic fermentation.

In our observation, *L. crispatus* was negatively correlated to simple sugars (i.e.: glucose, fructose) that, then again, were positively correlated to some taxa typical of CST IV (e.g.: *Streptococcus*, *Dialister*, *Prevotella*): these correlations suggest a possible nutrient competition with *Lactobacillus* species resulting in a stimulated CST IV elements’ growth favored by the higher pH following the reduced lactic fermentation. Additionally, it was previously reported that the vaginal fluids collected from women with bacterial vaginosis (BV) had a higher concentration of glucose (Vitali et al., 2015). Therefore, the absence of lactobacilli, especially those that are more effective at fermenting glucose, is linked to a higher availability of glucose in the vaginal environment, which can then encourage the growth of undesirable microorganisms, including bacteria linked to a dysbiotic environment (i.e., CST IV) (Navarro et al., 2023).

Interesting findings emerged as well when examining the associations between dietary data and the vaginal microbiota. Regardless of any confounders, we discovered that a higher animal protein balance (mostly derived from red and processed meat) in relation to the other macronutrients was substantially positively correlated with CST IV. To our knowledge, there are no prior studies reporting a positive independent association between increased animal protein intake and vaginal microbiota dysbiosis. However, we found some similarities between our study and previous research. One study showed that higher consumption of animal-sourced proteins was positively correlated with a higher Nugent score (indicating dysbiosis), in a cohort of Caucasian pregnant women, although no control was performed for confounders (Dall’Asta et al., 2021). Another study indicated that, even after adjusting for confounders, BV was linked to unhealthy dietary patterns, including also red and visceral meat (Noormohammadi et al., 2022). On the other hand, in two other studies of reproductive-age women, protein intake was not associated with molecular BV in multivariate analysis (Neggers et al., 2007; Shivakoti et al., 2020).

Although further research is needed to determine the underlying causes of the detrimental effects of a diet high in animal-sourced proteins, we can hypothesize an effect similar to that described for the gut microbiota. For instance, in the gut, a higher intake of plant-sourced protein is associated with greater abundance of “health-related” microorganisms (e.g., *Bifidobacterium*, *Roseburia*, *Lactobacillus*), as opposed to *Bacteroides* and *Clostridia*, which are mainly found primarily in the case of animal-sourced proteins (Prokopidis et al., 2020). From a biological point of view, we can speculate that a high consumption of animal proteins, particularly derived from red and processed meat, may increase the levels of inflammatory markers, which, in turn, may alter the balance of the microbiota environment (Bolte et al., 2021). Furthermore, animal protein fermentations may produce potentially toxic metabolites like ammonia and sulfides, that might favor the growth of harmful bacteria through increasing the vaginal pH (Windey et al., 2012).

We also pointed out that alcohol consumption was significantly positively associated with a BV condition (i.e., CST IV) and, in particular, was particularly positively correlated with the levels of *Gardnerella* spp. and *Ureaplasma* spp. Despite the limited number of studies in this area, it was observed that alcohol consumption may have a similar effect on the vaginal microbiota as tobacco, thereby increasing the risk of BV (Froehle et al., 2021; Turpin et al., 2021; Morsli et al., 2024). Alcohol could favor infections, due to its association with an alteration of the immune system function in an animal model (Loganantharaj et al., 2014). In a similar way, researchers have underscored a correlation between alcohol use and alterations in the oral microbiota, characterized by a diminished *Lactobacillales* abundance and an increased presence of *Neisseria*, *Streptococcus*, and *Prevotella* (Rajasekaran et al., 2024). The mechanisms of the negative effects of alcohol on the vaginal microbiota are still unknown; however, we can hypothesize both a direct effect on bacteria and an indirect effect on tissues, leukocyte function, and cytokine production, creating an environment conducive to microbial proliferation. Moreover, alcohol was found positively related to proline, an amino acid involved in collagen synthesis, which was also related to *Megasphaera* abundance. This is similar to what was reported in a recent study by Li and colleagues showing that, in the vaginal microecology, the high expression of the enzyme prolyl aminopeptidase (specifically releasing the terminal proline residue from a peptide) in BV-associated bacteria could be substantially correlated to the occurrence and the development of vaginal inflammatory diseases (Li et al., 2024).

We also noticed a beneficial effect of α-linolenic acid, one of the essential omega-3 polyunsaturated fatty acids mainly derived from plant sources such as nuts and seeds. Indeed, an increase of linolenic acid balance, relative to the other nutritional components, was inversely associated with CST III, dominated by the transitional species *L. iners*. The observed positive correlation between linolenic acid and *L. crispatus*, the hallmark of vaginal eubiosis, went in the same direction.

Although there are no data on the specific effects of omega-3 on the vaginal microbiota composition, numerous studies have shown that omega-3 fatty acids affect the gut microbial composition in different ways, including (i) a direct modulation of the gut microbial communities, (ii) an alteration of the inflammatory mediators, (iii) a regulation of the levels of SCFAs. By the means of these mechanisms, it has been demonstrated that the dietary intake of omega-3 fatty acids leads to the reduction of *Enterobacterales*, supporting the growth of *Bifidobacteria*, with an amelioration of intestinal inflammation and a higher protection from intestinal infections (Kumar et al., 2022). Interestingly, omega-3 fatty acid supplementation has already been observed to have beneficial effects on tryptophan neuroprotective metabolites during endurance training (Tomczyk et al., 2024) and, in an animal experimental model, Wang and colleagues observed that plant-derived α-linolenic acid visibly changed the initial proportion of vaginal microbiota bacteria by increasing *Lactobacillus*, *Faecalibacterium*, and *Parabacteroides*, as well as decreasing *Streptococcus* (Wang et al., 2020).

Moreover, lactic-acid bacteria (LAB) as lactobacilli, are able to transform α-linolenic acid into conjugated linoleic acids, which later get hydrogenated to saturated fatty acids such as stearic acid, thereby reducing the composition of PUFA (Kumar et al., 2022).

We also observed that total carbohydrates, vegetable proteins, total fiber and starch were negatively correlated with *Gardnerella* spp., possibly implying that their reduced consumption favors pathogens proliferation. We can therefore speculate that diets including a high intake of total carbohydrates and starch may lead to high levels of vaginal glycogen, which, in turn, might create a favorable environment for a lactobacilli-dominated flora with low levels of BV-associated microbes, as *Gardnerella* spp., as also reported by previous works (Miller et al., 2016; Song et al., 2020). Interestingly, starch was positively correlated to lactate, the main product of lactic fermentation, and to phenylalanine and glutamate, both within the neurotransmitter class.

Finally, even though it has been shown that the adherence to the Mediterranean diet can cause multiple changes in the gut microbiota (Meslier et al., 2020; Dahl et al., 2020), we did not observe a significant effect of this factor on the vaginal environment. Previous studies have shown that higher adherence to the Mediterranean diet (MEDI-LITE score ≥13) is associated with more pronounced beneficial effects, including reduced inflammation markers (Sureda et al., 2018), improved metabolic parameters (Galilea-Zabalza et al., 2018), and more substantial changes in gut microbiota composition (Meslier et al., 2020). Considering that in our population the mean MEDI-LITE score was 11 (range: 6-16) out of a possible maximum of 18, we can speculate that a higher adherence to the Mediterranean diet may be necessary to observe significant effects. Therefore, the moderate adherence observed in our population may explain the limited impact on the vaginal microbiota, suggesting that there may be a threshold effect where a more stringent dietary adherence is required to induce significant changes in vaginal microbial communities.

Overall, our data shed new light on the importance of the interaction between the gastrointestinal-tract and the vagina, the so-called ‘vagina-gut axis’, described for the first time by Ravel and Brotman in 2016 (Ravel and Brotman, 2016). A healthy diet could preserve the vaginal homeostasis by regulating the trafficking of bacterial species across the vagina and gut (bacterial translocation), in turn modulating the level and type of metabolites produced by the microbiota, acting as indirect players of the vagina-gut axis (Takada et al., 2023). In conclusion, we highlighted that specific dietary habits (i.e., reduced consumption of alcohol and animal proteins, higher intake of linolenic acid) can have a beneficial impact on the vaginal environment, through the maintenance of a microbiota mostly dominated by ‘protective’ *Lactobacillus* species such as *L. crispatus, L. gasseri*, and *L. jensenii.*

The main strengths of the present work include (i) the use of a standardized and validated tool to collect dietary data, (ii) a deep analysis of the vaginal ecosystem (i.e., classification of the vaginal microbiota composition in CST, quantification of vaginal metabolites) (iii) the extensive socio-demographic and behavioral data collection from the participants. Finally, to the best of our knowledge, this is the first study to use the CoDA (compositional data analysis) statistical technique to investigate the effect of macronutrient intake on the vaginal microbiota. This statistical method provides fully adjusted isocaloric estimates of the effects of specific nutrient balances on the vaginal microbiota, taking into account the compositional nature of the dietary data itself and being able to capture the interdependent dynamics of dietary components.

We are fully aware of some limitations of this study. The main one is the cross-sectional design, which limits causal inference for the association between diet and CST, and reverse causation cannot be excluded, particularly because the study does not allow us to determine whether dietary behaviors preceded or followed the changes in vaginal bacterial composition. In addition, the voluntary participation of young and highly educated women in the study suffers from an inherent selection bias, which limits the generalizability of our results to older and less educated adult women, because the sample was self-selected and not fully representative of the Italian population. However, this selection bias is likely to affect our results toward the null, thus underestimating the observed associations. Finally, although we controlled for known potential confounders, we cannot rule out the possibility of residual confounding due to unmeasured factors (e.g. physical activity, sexual habits). However, we speculate that our participants were relatively healthy and had a good lifestyle, supported by the fact that they were volunteers, young medical students, mostly non-smokers, and light drinkers (Fry et al., 2017). In terms of sexual behavior, as in previous studies (Duberstein Lindberg and Singh, 2008; Exavery et al., 2015; Jackson et al., 2019), we assumed that single women were more likely to have a higher number of partners than married/cohabiting women. We accounted for this by controlling for marital status, used as a proxy for sexual behavior.

Thus, more research is required to better understand the mechanisms underlying the impact of dietary macronutrients on the vaginal ecosystem as well as the role and activity of saturated and unsaturated fatty acids on the vaginal environment, and how the vaginal inhabitants may contribute to their metabolism and transformation. In this context, even though it is crucial to assess long-term dietary patterns when investigating the potential effects of dietary habits on the microbiome, future studies could benefit from combining the FFQ with either 24h recall or 3-day food diary to provide additional insights into the dietary habits of the enrolled cohort.

In addition, future *in vitro* experimental studies will be useful to demonstrate a beneficial or detrimental effect of specific dietary metabolites on the inhabitants of the vaginal ecosystem.

## Data Availability

The datasets presented in this study can be found in online repositories or in the **Supplementary Material**. The names of the repository/repositories and accession number(s) can be found in the article (see section 2.7).
