# Supplementary material for: Dietary habits and vaginal environment: can a beneficial impact be expected?
Source: Front Cell Infect Microbiol. 2025 Jun 18;15:1582283. doi: 10.3389/fcimb.2025.1582283 (PMC12213695; doi:10.3389/fcimb.2025.1582283)
Supplement: Supplementary file 3 [file DataSheet3.docx]

Dietary habits and vaginal environment: can a beneficial impact be expected?

Marielle Ezekielle Djusse^1,2*^, Federica Prinelli^3*^, Tania Camboni^3^, Camilla Ceccarani^3,4^, Clarissa Consolandi^3,4^, Silvia Conti^3,5^, Margherita Dall’Asta^6^, Francesca Danesi^7,8^, Luca Laghi^7,9^, Francesco Matteo Curatolo^6^, Sara Morselli^1^, Claudio Foschi^1,10^^, Paola Castellano^11^, Antonella Marangoni^1$^, Marco Severgnini^3,4$^

^*^These two authors contributed equally to this work and share first authorship

^$^These two authors contributed equally to this work and share last authorship

^1^Section of Microbiology, Department of Medical and Surgical Sciences (DIMEC), Alma Mater Studiorum - University of Bologna, Bologna, Italy.

^2^International PhD College, Collegio Superiore of Alma Mater Studiorum, University of Bologna, Bologna, Italy.

^3^Institute of Biomedical Technologies, National Research Council, Segrate, Italy.

^4^National Biodiversity Future Center S.c.a.r.l., Palermo, Italy.

^5^Department of Medical Sciences, University of Ferrara, Ferrara, Italy.

^6^Department of Animal Science, Food and Nutrition (DIANA), Università Cattolica Del Sacro Cuore, Piacenza, Italy.

^7^Human Nutrition Unit, Department of Agricultural and Food Sciences (DISTAL), University of Bologna, Cesena, Italy.

^8^Interdepartmental Centre for Agri-Food Industrial Research (CIRI Agrifood), University of Bologna, Cesena, Italy.

^9^Centre of Foodomics, Department of Agricultural and Food Sciences (DISTAL), University of Bologna, Cesena, Italy.

^10^Microbiology Unit, IRCCS Azienda Ospedaliero-Universitaria di Bologna, Bologna, Italy.

^11^ Department of Medical and Surgical Sciences (DIMEC), Alma Mater Studiorum - University of Bologna, Bologna, Italy.

^^^Corresponding author: Claudio Foschi

# TABLE OF CONTENTS

[SUPPLEMENTARY FIGURES 3](#_bookmark0)

[Figure S1 3](#_bookmark1)

[Figure S2 3](#_bookmark2)

[Figure S3 4](#_bookmark3)

[Figure S4 5](#_bookmark4)

[Figure S5 6](#_bookmark5)

[SUPPLEMENTARY TABLES 7](#_bookmark6)

[Table S1 7](#_bookmark7)

Table S2 8

SUPPLENTARY EXCEL FILES………………………………………………………………………………………………………………………………………………9

Excel File S1…………………………………………………………………………………………………………………………………………………………….9

Excel File S2…………………………………………………………………………………………………………………………………………………………….9

2 / 7

# SUPPLEMENTARY FIGURES

**Figure S1.** Stacked bars of the relative abundance of bacterial taxa over all the samples (n=113). Only the main taxa (average abundance >0.4% over all the samples) are represented here, with Lactobacillus genus further subclassified to species level. Less abundant taxa are grouped in the “Other” category. Colors below the single sample represent the CST in both the traditional 5-levels classification and the 3-level one used here (i.e.: grouping together CST I, II and V)


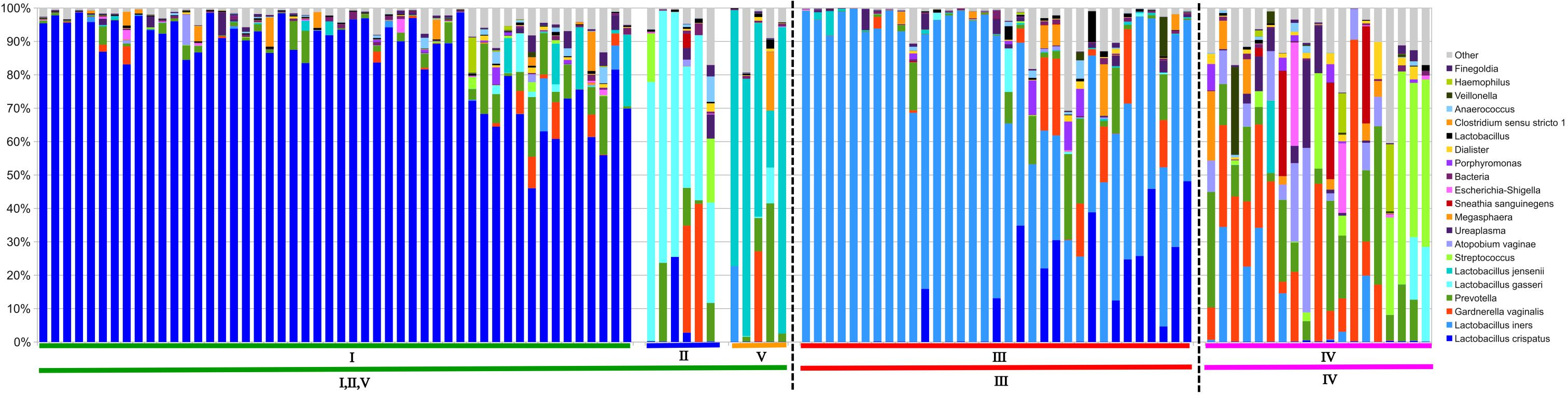


**Figure S2.** (A) Boxplot of alpha-diversity over CST estimated through the “observed species” metric. Box and whiskers plot depict the median (red line) and the first and third quartile of the distribution, whereas points are single samples; (B) principal coordinate analysis (PCoA) based on the unweighted UniFrac distance among samples, illustrating the clustering of vaginal microbiota samples according to CST classification. Each point represents a sample, colored according to the CST, centroids are the average of the coordinates and ellipses represent the SEM-based confidence interval. The second and third principal coordinates are represented.


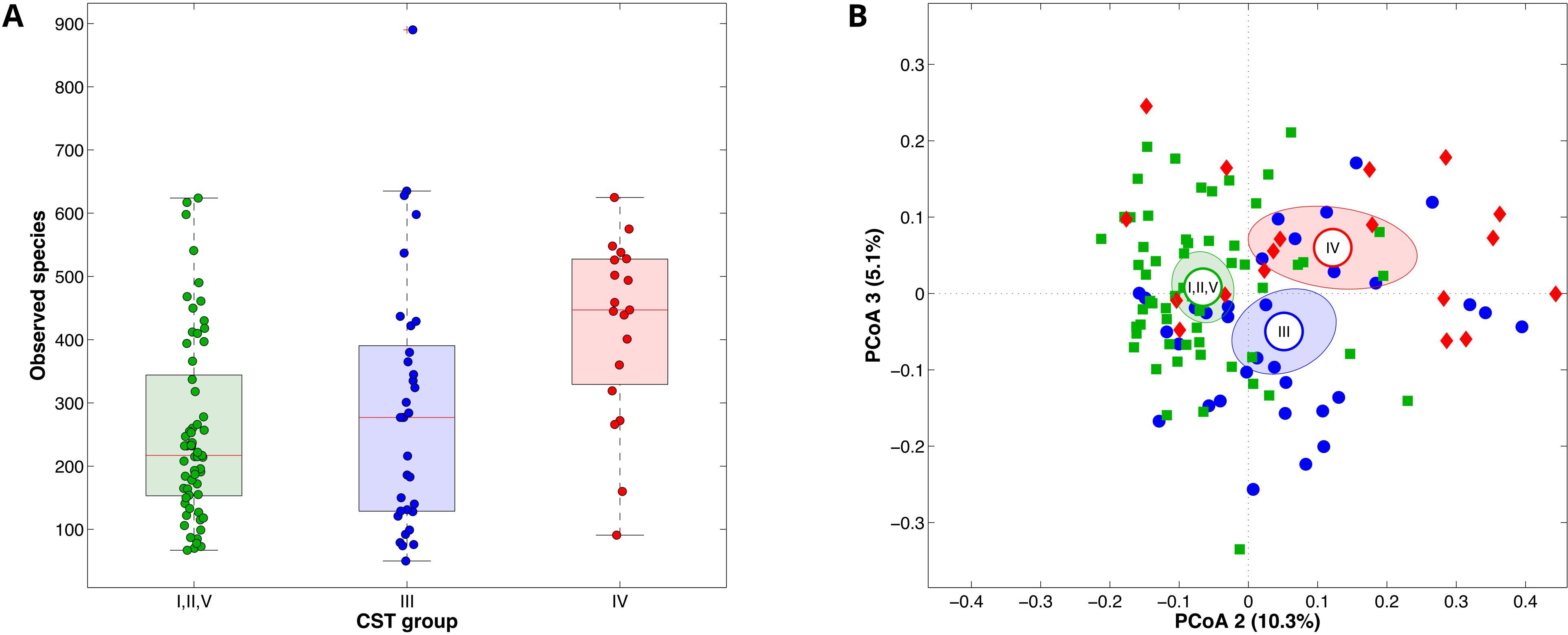


3 / 7

**Figure S3**. Spearman’ correlation between macronutrients and bacteria composition at genus level (n=113). Black dots indicate significant correlations (p-value<0.05)


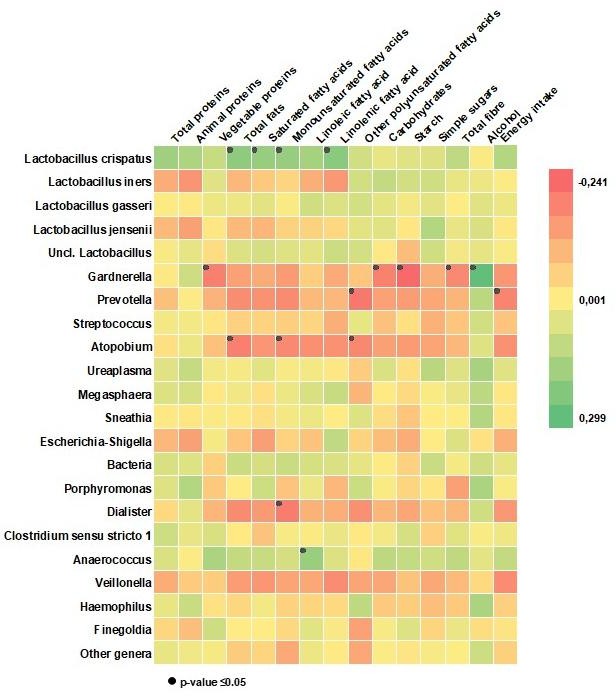


4 / 7

**Figure S4**. Spearman’ correlation between metabolites and bacteria composition at genus level (n=113). Black dots indicate significant correlations (p-value<0.05)


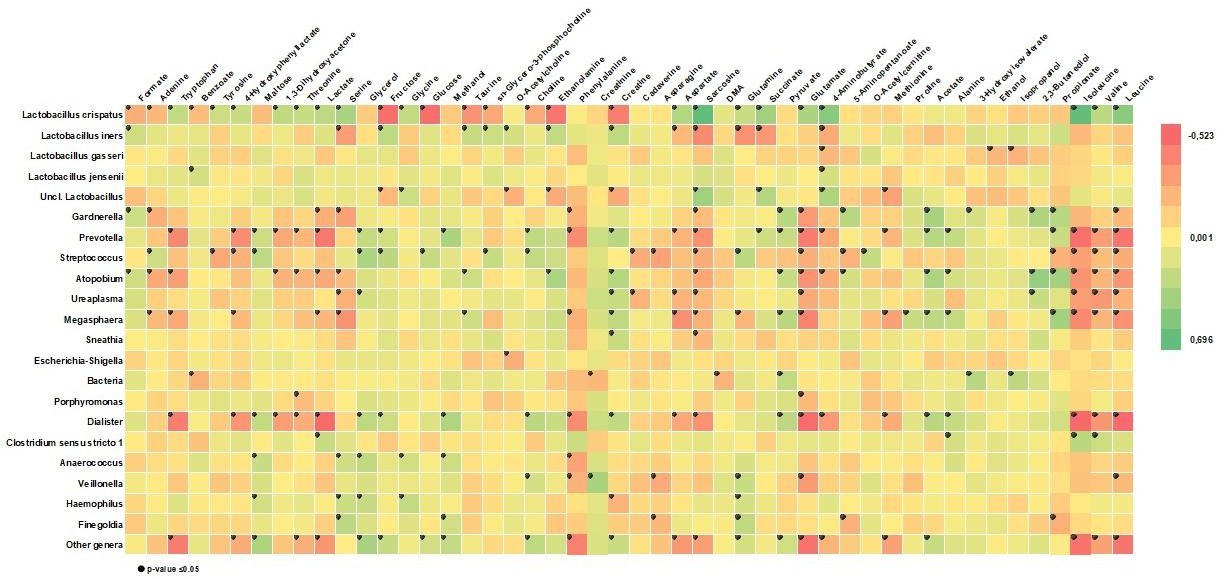


**Figure S5**. Spearman’ correlation between metabolites and macronutrients (n=113). Black dots indicate significant correlations (p-value<0.05)


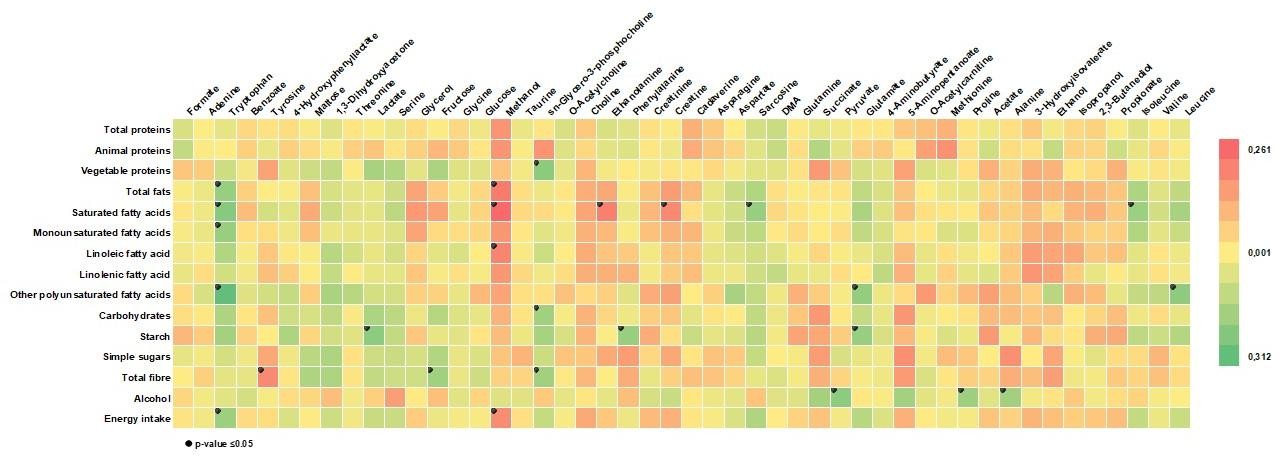


6 / 7

# SUPPLEMENTARY TABLES

**Table S1*.*** Multinomial logistic regression coefficient (β-coefficient) and standard errors (SE) of CST in relation to the macronutrient balances, total energy, fibre and alcohol intake.

|  | **CST III**  (n=33, 29.20%) | |  | **CST IV**  (n=19, 16.81%) | |  |
| --- | --- | --- | --- | --- | --- | --- |
|  | **β-coefficient** | **SE** | **p-value** | **β-coefficient** | **SE** | **p-value** |
| Total fibre (1 gram increase) | 0.170 | 0.120 | 0.157 | 0 .174 | 0.133 | .190 |
| Alcohol (1 AU increase) | 0.934 | 0.633 | 0.140 | 1.570 | 0.777 | .043 |
| Energy intake (1 Kcal increase) | -0.003 | 0.001 | 0.109 | -0.002 | 0.002 | .271 |
| *Macronutrients balances* |  |  |  |  |  |  |
| Animal proteins vs other macronutrients | -0.566 | 1.031 | 0.583 | 2.702 | 1.268 | .033 |
| Vegetable proteins vs other macronutrients | -1.635 | 3.593 | 0.649 | -3.608 | 3.961 | .362 |
| Simple sugars vs other macronutrients | 1.590 | 1.036 | 0.125 | 0.690 | 1.084 | .525 |
| Starch vs other macronutrients | 1.670 | 2.331 | 0.474 | 1.668 | 2.492 | .503 |
| Saturated fatty acids vs other macronutrients | -0.991 | 1.820 | 0.586 | -0.817 | 2.142 | .703 |
| Monounsaturated fatty acids vs other macronutrients | 2.630 | 1.861 | 0.158 | -0.423 | 1.851 | .819 |
| Linoleic fatty acid vs other macronutrients | 1.801 | 1.725 | 0.296 | 1.732 | 1.732 | .317 |
| Linolenic fatty acid vs other macronutrients | -5.320 | 1.955 | 0.007 | -1.176 | 1.846 | .524 |
| Other polyunsaturated fatty acids vs other macronutrients | 0.821 | 0.497 | 0.099 | -0.767 | 0.526 | .145 |
| MEDI-LITE° | 0.170 | 0.115 | 0.140 | 0.115 | 0.135 | 0.395 |

Standard error (SE).

Reference category for the regression model was CST I, II, and V.

The model also included terms for age, BMI, marital status, and hormonal contraception use.

°The model did not include terms for total fibre and alcohol intake.

7/7

**Table S2*.*** Multinomial logistic regression coefficient (β-coefficient) and standard errors (SE) of CST in relation to the macronutrient balances, total energy, fibre and alcohol intake adjusted for psychological distress (PSS scale; available for 103 women)

|  | **CST III** | |  | **CST IV** | |  |
| --- | --- | --- | --- | --- | --- | --- |
|  | **β-Coefficient** | **SE** | **p value** | **β-Coefficient** | **SE** | **p value** |
| Total fibre (1 gram increase) | .2556247 | .1433539 | 0.075 | .2443676 | .1494679 | 0.102 |
| Alcohol (1 AU increase) | 1.119419 | .6753767 | 0.097 | 1.622408 | .7898965 | 0.040 |
| Energy intake (1 Kcal increase) | -.0038843 | .0019948 | 0.052 | -.0030921 | .0021496 | 0.150 |
| *Macronutrients balances* |  |  |  |  |  |  |
| Animal proteins vs other macronutrients | .0552169 | 1.116714 | 0.961 | 2.947684 | 1.372236 | 0.032 |
| Vegetable proteins vs other macronutrients | -.612895 | 3.906558 | 0.875 | -5.050278 | 4.323231 | 0.243 |
| Soluble sugars vs other macronutrients | 1.892724 | 1.107079 | 0.087 | 1.356281 | 1.170195 | 0.246 |
| Starch vs other macronutrients |  |  |  |  |  |  |
| Saturated fatty acids vs other macronutrients | -.3357106 | 2.070374 | 0.871 | -.7955899 | 2.310891 | 0.731 |
| Monounsaturated fatty acids vs other macronutrients | 2.160048 | 2.017636 | 0.284 | -.0823618 | 1.927009 | 0.966 |
| Linoleic fatty acid vs other macronutrients | 2.200344 | 1.901759 | 0.247 | 1.736833 | 1.913183 | 0.364 |
| Linolenic fatty acid vs other macronutrients | -7.443688 | 2.314815 | 0.001 | -2.021834 | 1.999852 | 0.312 |
| Other polyunsaturated fatty acids vs other macronutrients | .904346 | .5667165 | 0.111 | -.6249929 | .5570733 | 0.262 |

Standard error (SE).

Reference category for the regression model was CST I, II, and V.

____________________________________________________________________________________________________

8/8

# SUPPLEMENTARY EXCEL FILES

# Excel File S1. Raw metabolomic data (see Excel File attached)

# Excel File S2. Raw nutritional data (see Excel File attached)

# ____________________________________________________________________________________

9/9
